# Why Is Non-suicidal Self-injury More Common in Women? Mediation and Moderation Analyses of Psychological Distress, Emotion Dysregulation, and Impulsivity

**DOI:** 10.1080/13811118.2022.2084004

**Published:** 2022-06-13

**Authors:** Nina M. Lutz, Sharon A. S. Neufeld, Roxanne W. Hook, Peter B. Jones, Edward T. Bullmore, Ian M. Goodyer, Tamsin J. Ford, Samuel R. Chamberlain, Paul O. Wilkinson

**Keywords:** Distress, emotion regulation, gender, impulsivity, NSSI, self-harm

## Abstract

**Objective:**

Non-suicidal self-injury (NSSI) appears to be more common among women than men, though the underlying reasons for this remain unclear. In a community sample of young adults *(N¼* 996, aged 18–33) assessed during the COVID-19 pandemic, we investigated alternative explanation for the NSSI prevalence gap: are women more likely to experience the feelings which lead to NSSI as a coping strategy, or does this prevalence gap result from differences in how men and women respond to distress?

**Methods:**

Cross-sectional mediation and moderation analyses tested how self-reported psychological distress (K10), emotion dysregulation (DERS), and impulsivity (UPPS-P) may contribute to a higher prevalence of NSSI among women.

**Results:**

Women were twice as likely as men to report past-year NSSI (14.47% versus 7.78%, OR = 2.00, 95% CI [1.29, 3.13]). Women reported significantly higher psychological distress and significantly lower sensation seeking and positive urgency than men. Psychological distress partially statistically mediated the relationship between gender and past-year NSSI. Gender did not significantly moderate associations between psychological distress, emotion dys-regulation, or impulsivity and past-year NSSI. Past-year NSSI prevalence did not significantly decrease with age and we found no significant age by gender interaction.

**Conclusions:**

Greater levels of NSSI in young women are partly explained by their greater levels of psychological distress, but not by differences in how men and women respond to this distress. Given similar levels of psychological distress, emotion dysregulation, and impulsivity, women and men are similarly likely to experience NSSI.

## Introduction

Non-suicidal self-injury (NSSI) is the direct and deliberate damage of the body without suicidal intent (International Society for the Study of Self-Injury, 2018). The literature on NSSI is heavily gendered; research has focused on young women and girls, reinforcing assumptions of self-injury as a “feminine” behavior (Brickman, 2004; Chandler, Myers, & Platt, 2011; Millard, 2013). As a result, NSSI in men is poorly understood. Reports of gender differences in NSSI prevalence vary substantially across studies but meta-analytic findings indicate that women are significantly more likely than men to report NSSI (Bresin & Schoenleber, 2015). However, research has yet to investigate the underlying reasons for this.

In the present study, we explore alternative explanations for the NSSI prevalence gap. First, women may be more likely than men to experience feelings which precipitate NSSI. Though the behavior can be driven by a range of motivations, it is most often used to alleviate negative affect (Edmondson, Brennan, & House, 2016; Taylor et al., 2018). Feelings of sadness, emptiness, hopelessness, and anxiety commonly precede NSSI and decrease afterwards (Armey, Crowther, & Miller, 2011; Klonsky, 2009). Furthermore, internalizing symptomology is associated with NSSI in both clinical and non-clinical samples (Cipriano, Cella, & Cotrufo, 2017; Valencia-Agudo, Burcher, Ezpeleta, & Kramer, 2018). Women are more likely than men to experience depression and anxiety and this gender difference is largest in mid-adolescence (Salk, Hyde, & Abramson, 2017; Steel et al., 2014). This parallels recent findings that the NSSI gender prevalence gap is greatest during mid-adolescence due, in part, to adolescent girls’ significantly higher levels of psychological distress (Wilkinson et al., 2022). Higher levels of depressive and anxious symptoms—which can be measured as psychological distress (Kessler et al., 2002)—may therefore contribute to women’s greater prevalence of NSSI.

Second, the prevalence gap may be due to differences in how women and men respond to distress. Emotion regulation encompasses the automatic and purposeful processes of managing emotional responses, including initial awareness and acceptance of emotions, the ability to control behavior while experiencing strong emotion, and the use of appropriate emotion modulation strategies (Gratz & Roemer, 2004). Difficulties deploying effective emotion regulation strategies place individuals at particularly high risk of using NSSI to manage negative affect, however, each of these facets are significantly associated with NSSI (Wolff et al., 2019). Researchers have documented patterns of gender differences in emotion regulation processes and theorized that these differences, in turn, underlie gender differences in prevalence rates of many forms of psychopathology (Nolen-Hoeksema, 2012). Women tend to show more awareness and understanding of their emotions and engage in more emotion regulation strategies than men, but paradoxically, this increased attention can result in emotion dysregulation if their ways of responding prolong and exacerbate negative feelings (e.g., rumination) (Nolen-Hoeksema, 2012). People with high levels of emotion dysregulation are likely to experience their feelings as more intense and uncontrollable and may turn to NSSI to interrupt the “emotional cascade” (Nolen-Hoeksema, 2012; Selby, Anestis, & Joiner, 2008). Women endorse more emotion regulation functions of NSSI than men (Victor et al., 2018; Whitlock et al., 2011), suggesting that greater emotion dysregulation may contribute to women’s high prevalence of NSSI.

Finally, since its earliest appearances in the literature, self-injury has been described as “impulsive” (Burnham, 1969; Favazza & Conterio, 1989) and impulsivity has been central to academic explanations of women’s reasons for self-harming (Chandler et al., 2011). Impulsivity is multifaceted (Smith et al., 2007) and empirical support for associations between disparate dimensions of impulsivity and NSSI is mixed. However, it appears that negative urgency—a tendency to act rashly while experiencing negative emotion, which is conceptually similar to emotion dysregulation—is especially relevant to NSSI (Lockwood, Daley, Townsend, & Sayal, 2017, Lockwood, Townsend, Allen, Daley, & Sayal, 2020). Trouble sticking with difficult tasks and low planning may be specifically associated with recurrent NSSI in those who want to stop (Lockwood et al., 2017; Riley, Combs, Jordan, & Smith, 2015). Cessation as a long-term goal requires persevering through unpleasant feelings and urges to perform NSSI whilst committing to alternative coping strategies. Women and men appear to exhibit similar levels of impulsivity across these NSSI-relevant domains (Argyriou, Um, Wu, & Cyders, 2020; Cyders, 2013). However, given gendered assumptions about the role of impulsivity in NSSI (Chandler et al., 2011), research is needed to clarify whether impulsivity contributes to the NSSI prevalence gap.

In the present study, we hypothesized that past-year NSSI would be more common in women than men. Should this be the case, we then planned to test two sets of competing (though not mutually exclusive) hypotheses to explain the gender gap. First, psychological distress, emotion dysregulation, or impulsivity may *mediate* the relationship between gender and past-year NSSI; the NSSI gender gap may be accounted for by different levels of these traits in women versus men. Second, gender may *moderate* the effects of psychological distress, emotion dysregulation, or impulsivity on past-year NSSI; these traits may be more strongly related to NSSI in women than in men. Greater NSSI in women may result from a combination of these effects. Based on evidence that women experience greater levels of distress (Salk et al., 2017) and NSSI is typically a response to distress (Edmondson et al., 2016; Lockwood et al., 2020), our primary hypothesis was that psychological distress would significantly mediate the association between gender and NSSI, as recently shown in an adolescent sample (Wilkinson et al., 2022). The remaining analyses in this population sample of young adults were exploratory. In addition, we expected the prevalence of past-year NSSI to decrease with age (Daukantaitė et al., 2021; Moran et al., 2012; Plener, Schumacher, Munz, & Groschwitz, 2015), and we planned to ascertain whether age moderates the gender difference in past-year NSSI prevalence.

## Methods

### Participants

Data were collected May–July 2020 as part of a study assessing mental health during the COVID-19 pandemic within the NeuroScience in Psychiatry Network (NSPN) cohort. Participants were 996 young adults aged 18 to 33 years (M = 25.54, *SD* = 3.10). In response to the question “What is your gender?,” most participants identified as female (n = 629, 63.15%) or male (n = 360, 36.14%), hereafter referred to as “women” and “men.” Seven participants reported their gender as “other.” Most participants (71.29%) had attained an undergraduate degree, postgraduate degree, or professional qualification; 78.01% reported their ethnicity as white, 9.84% Asian, 3.82% Black, 6.33% mixed or multiple ethnic groups, and 1.41% another ethnicity. These rates are comparable to the baseline NSPN cohort, which is broadly representative of ethnicity in the general population of England and Wales (Kiddle et al., 2018).

### Procedure

The NSPN cohort is a general population sample of 2,403 individuals across Cambridgeshire and Greater London, aged 14 to 24 at baseline, established in 2012 to longitudinally investigate both healthy and psychopathological development. NSPN participants were originally recruited via invitations sent by primary care physicians, schools and Further Education colleges, and by purposive advertisement. Baseline cohort characteristics, as well as previous follow-up data, have been previously described elsewhere (Kiddle et al., 2018).

To recruit the present 2020 sample, emails were sent to all 2,036 participants who had not withdrawn from NSPN. Responses were received from 1,005. An additional 22 participants from a separate NSPN depression cohort were invited, for an initial sample of 1,027. Of the 1,027, six returned submissions with no responses; five reported an age inconsistent with baseline NSPN data, suggesting the survey had been completed by a parent (i.e., participant may have provided their parents’ contact details rather than their own); a further 19 did not respond to the NSSI measure; and one did not report age or gender. Thus, we had a final sample of 996 young adults, of whom 21 came from the depression cohort.

Compared to NSPN participants who did not participate in this follow-up, those who did were significantly older (*p* = .004) and more likely to identify as female (*p* < .001). There were no differences in ethnicity (*p* = .53), baseline levels of psychological distress (*p* = .56), or baseline prevalence of past-year NSSI (*p* = .09).

Ethical approval for the current study was provided by the Cambridge East Research Ethics Committee (reference number 16/EE/0260, project: *Neuroscience in Psychiatry Network (NSPN) COVID-19 mental wellbeing and impulsivity follow-up study*, Chief Investigator SRC) following the Declaration of Helsinki. Participants provided electronic informed consent and were compensated £25.

## Materials

### Drugs, Alcohol and Self-Injury Questionnaire (DASI)

NSSI history was assessed via a yes/no question from the DASI, with established validity and reliability (Wilkinson, Qiu, Neufeld, Jones, & Goodyer, 2018): *“Have you ever tried to hurt yourself on purpose without trying to kill yourself? (for example: things like burning, cutting or scratching yourself).” “* Participants who responded yes were then asked how many times they had hurt themselves in the past month, past year, since turning 18, and in their entire life. The past-year NSSI group included participants who reported at least one instance of NSSI within the past month or past year.

### Kessler Psychological Distress Scale (K10)

The K10 includes ten questions assessing anxious and depressive symptoms (e.g., feeling nervous, hopeless, tired, restless, sad, worthless) (Kessler et al., 2002). Participants reported “how much of the time” they felt this way in the past month on a five-point scale from “none of the time” to “all of the time.” Higher sum scores indicate greater psychological distress and can be used to screen for anxiety and mood diagnoses (Kessler et al., 2002). The K10 demonstrates consistent psychometric properties across women and men and can therefore be used to compare gender differences (Baillie, 2005; Kessler et al., 2002). Internal consistency in the present sample was high (Cronbach’s α = 0.91).

### Difficulties in Emotion Regulation Scale (DERS)

Participants were presented with statements about how they respond to their emotions (e.g., “I am confused about how I feel,” “When I’m upset, I have difficulty concentrating”) and reported how much of the time they felt that way on a five-point scale from “almost never” to “almost always.” The 18-item short form used here (Victor & Klonsky, 2016) generates six sub-scores capturing different components of trait emotion dysregulation: awareness of emotions, clarity about emotions, acceptance of emotions, access to effective emotion regulation strategies, ability to engage in goal-directed behavior while upset, and ability to manage impulses while upset. Higher scores reflect more dysregulation. Here, we utilize the DERS total score since all subscales have been shown to be comparably associated with NSSI (Wolff et al., 2019). The DERS demonstrates strong psychometric properties (Victor & Klonsky, 2016) and measurement invariance across men and women (Ritschel, Tone, Schoemann, & Lim, 2015). Internal consistency of the DERS total score in the present sample was high (Cronbach’s α = 0.91).

### UPPS-P Impulsive Behavior Scale

Participants were presented with statements about how they act and think (e.g., “I finish what I start,” “I quite enjoy taking risks”) and indicated how much they agreed with each statement on a four-point scale from “agree strongly” to “disagree strongly” (Cyders, Littlefield, Coffey, & Karyadi, 2014). The 20-item short form used here contains five trait impulsive subscales: *Negative Urgency*, the tendency to react rashly to negative emotions; *Lack of Perseverance*, a difficulty seeing thing through to the end; *Lack of Premeditation*, the tendency to act without thinking; *Sensation Seeking*, the tendency to pursue novel and thrilling experiences; and *Positive Urgency*, the tendency to react rashly to positive emotions. The UPPS-P demonstrates good construct validity (Smith et al., 2007) and measurement invariance across men and women (Cyders, 2013). Internal consistencies of the five subscales were acceptable or good (Cronbach’s α, Negative Urgency = 0.79, Lack of Perseverance = 0.68, Lack of Premeditation = 0.77, Sensation Seeking = 0.69, Positive Urgency = 0.81).

### Statistical Analysis

All analyses were conducted in STATA 14.2. Age and gender distributions between those with and without past-year NSSI were compared using a t-test and Fisher’s Exact Test.

Because our hypotheses focused on the prevalence gap between women and men, the seven participants who reported their gender as “other” were removed from primary analyses (there was not enough statistical power to compare this group to women and men). In separate univariate binary logistic regressions, we tested whether psychological distress (K10 score), emotion dysregulation (DERS total score), or impulsivity (UPPS-P subscales) were associated with NSSI (yes versus no past-year NSSI) and gender (female versus male).

Mediation and moderation analyses both help us understand the relationship between an independent and dependent variable: mediators explain how the two variables are related, while moderators affect the strength or direction of that association. Variables associated with both NSSI and gender were investigated as possible statistical mediators between gender and NSSI, using bootstrapped generalized structural equation models. Variables significantly associated with NSSI were included in binary logistic regression moderation analyses, with an interaction term between gender and each self-report measure.

Two multiple binary logistic regressions analyzed linear and quadratic interaction effects of age and gender on past-year NSSI. For descriptive purposes, age bins are used in Figure 2; however age was a continuous variable in regression analyses.

Given the timing of data collection, high NSSI rates could be related to worsening mental health during the COVID-19 pandemic (Vindegaard & Benros, 2020; Wang, Kala, & Jafar, 2020) and may not represent the population at normal times. We tested for this in a secondary analysis. Most participants (*n* = 548) were included in a previous follow-up study of the NSPN cohort in 2017–2018 (Chamberlain et al., 2019). Those data were collected when participants were aged 17-31 (*M* = 23.42 years), an average of 28.81 months prior to the data presented here.

## Results

### Prevalence of NSSI

Overall, 331 participants (31.12% of the sample) reported lifetime NSSI, of whom 125 (12.55% of the sample) reported past-year NSSI. Participants with or without past-year NSSI did not differ in age (t(994) = 1.17, *p =* .24). Gender distribution significantly differed by group (Fisher’s Exact Test, *p <* .001): 14.47% of women and 7.78% of men reported past-year NSSI, as did six of the seven participants (85.71%) who reported their gender identity as “other.” Past-year NSSI prevalence did not differ significantly between the 2017–2018 assessment and present 2020 assessment (McNemar’s Test, χ2(1, *n =* 548) = 2.85, *p =* .09; 13.50% pre-pandemic versus 10.77% during the pandemic).

### Univariate Associations of Putative Mechanistic Variables with NSSI and Gender

Univariate regressions (Table 1) showed the following significant associations with past-year NSSI: greater psychological distress, emotion dysregulation, negative urgency, lack of premeditation, and positive urgency, as well as lower sensation seeking. Female gender was significantly associated with greater psychological distress, while male gender was associated with greater sensation seeking and positive urgency. These three variables, associated with both gender and NSSI, were considered in the following mediation analyses.

### Mediation Analyses

**Figure 1:**
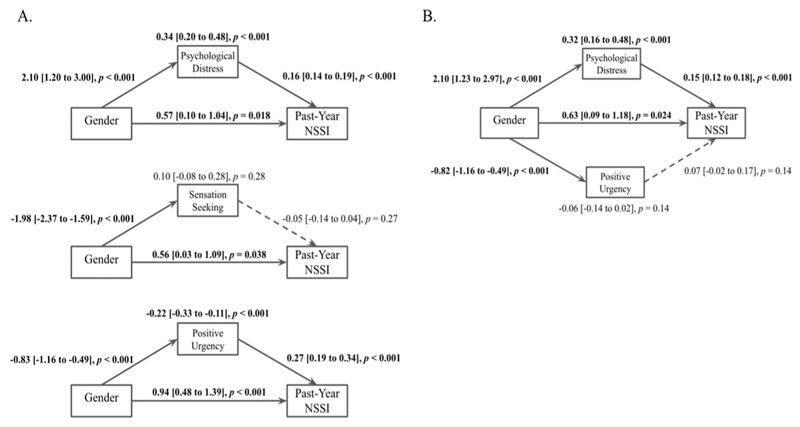
Path diagrams of the effect of gender (female or male) on past-year NSSI via psychological distress, sensation seeking, and positive urgency individually (A) and in a joint model including psychological distress and positive urgency (B). The models show standardized coefficients with 95% confidence intervals of direct effects between variables (displayed adjacent to each respective arrow) and indirect effects of gender on NSSI through each mediator (displayed above respective mediators). Bold text and solid lines indicate significance at *p <* .05.

Psychological distress significantly mediated the relationship between gender and pastyear NSSI (Figure 1): 37% of the effect of gender on past-year NSSI was explained via psychological distress. Sensation seeking was not a significant mediator. Positive urgency negatively mediated the effect of gender on past-year NSSI, since men reported greater positive urgency than women, and this urgency was related to increased NSSI. In a joint mediation model, only psychological distress showed a significant effect.

### Moderation Analyses

All analyses were non-significant: gender did not significantly moderate the effects of psychological distress (b = 0.002, 95%CI [−0.06,0.07], *p =* .95), emotion dysregulation (b = [−0.007, 95%CI [[−0.05,0.03], *p =* .74), negative urgency (b = [−0.03, 95%CI [[−0.21,0.16], *p =* .77), lack of premeditation (b = [−0.08, 95%CI [[−0.15,0.32], *p =* .50), sensation seeking (b = 0.05, 95%CI [[−0.13,0.22], *p =* .61), or positive urgency (b = 0.03, 95%CI [[−0.15,0.21], *p =* .76) on past-year NSSI.

### Age Effects, and Their Moderation by Gender

**Figure 2:**
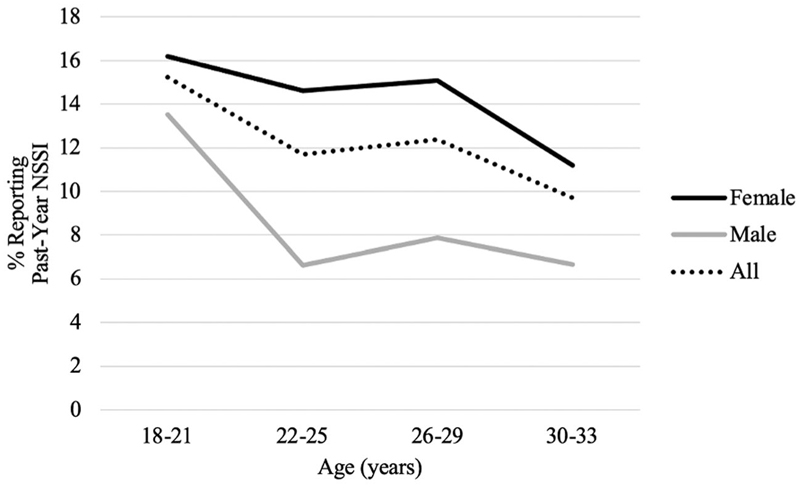
Effects of age and gender on prevalence of past-year NSSI.

Past-year NSSI prevalence appeared lower at older ages, but this was not statistically significant. The prevalence gap did not vary with age (linear and quadratic age x gender interactions non-significant; Table 2, Figure 2).

## Discussion

In this community sample of young adults, we investigated how psychological distress, emotion dysregulation, and impulsivity may contribute to a higher prevalence of NSSI among women. Our primary finding was that psychological distress statistically mediated the relationship between gender and NSSI - women were twice as likely as men to report past-year NSSI, with over a third of the variance explained by women’s significantly higher levels of distress. These findings support our first proposed explanation for the prevalence gap: NSSI is often used to cope with negative feelings associated with depression and anxiety (e.g., sadness, nervousness, worthlessness), and women are at higher risk of NSSI because they experience greater levels of these symptoms than men.

Empirically supported theory describes how individual (i.e., affective, biological, and cognitive predispositions), interpersonal (e.g., sexual violence), and sociocultural factors (e.g., structural gender inequality, media exposure) interact and contribute to higher levels of depressive symptoms and distress in women (Hyde & Mezulis, 2020). A greater tendency for rumination coupled with more adverse experiences appears to be particularly relevant, both significantly mediating women’s higher levels of psychological distress (Matud, Bethencourt, & Ibanez, 2015; Nolen-Hoeksema, 2012; Nurullah, 2010) and significantly contributing to NSSI risk (Voon, Hasking, & Martin, 2014). However, Hyde and Mezulis’ model emphasizes the equifinality of this distress, explaining that it results from numerous combinations of vulnerability and stress, and that there is no single explanation for women’s higher levels. The underlying reasons for higher prevalence of NSSI in women are likely to be similarly complex.

In addition to psychological distress, positive urgency and sensation seeking were identified as potential mediators. We found a negative statistically mediating effect of positive urgency on NSSI. Positive urgency likely weakened the overall association between gender and NSSI because men displayed higher positive urgency, which was associated with NSSI, yet women were more likely to report NSSI. Researchers have begun to investigate the relationship between gender, positive urgency, and NSSI (Peckham et al., 2020) based on evidence that men report higher positive urgency than women (Cyders, 2013) and more risky behaviors while experiencing positive affect (Cyders & Smith, 2010). However, most literature has focused on NSSI in the context of negative emotions. Future research should elucidate whether impulsivity while experiencing positive affect directly contributes to NSSI engagement, and whether this is particularly relevant to NSSI among men.

The relationship between sensation seeking and NSSI is also ambiguous. Men reported significantly higher levels than women, consistent with past work identifying sensation seeking as one of the few psychological traits to show meaningful gender differences (Argyriou et al., 2020; Hyde, 2014). However, in contrast with previous studies (Lockwood et al., 2017), we found that NSSI was significantly associated with lower, rather than higher, sensation seeking. We might therefore expect lower levels of sensation seeking in women to contribute to their higher rates of NSSI in this sample, yet the mediation analysis was non-significant. Many people describe self-injuring to generate exhilaration or excitement (Edmondson et al., 2016), suggesting a direct connection between sensation seeking and NSSI. Additional research clarifying the relationship is needed.

We found no evidence for our second explanation of the prevalence gap; women are not more likely to engage in NSSI due to differences in their response to distress. Levels of emotion dysregulation and negative urgency did not differ by gender, and all moderation analyses were non-significant—at similar levels of psychological distress, emotion dysregulation, and impulsivity, men and women are similarly likely to engage in NSSI. Consistent with this, UPPS-P subscales are similarly associated with risky behaviors across genders (Cyders, 2013), and gender does not moderate the relationships between emotion dysregulation, positive urgency, or negative urgency and NSSI (Peckham et al., 2020; Wolff et al., 2019). Furthermore, longitudinal associations between depressive symptoms and NSSI does not differ between adolescent boys and girls (Tilton-Weaver, Marshall, & Svensson, 2019). We have found that psychological distress, emotion dysregulation, and impulsivity, which have shown significant associations with NSSI in primarily female samples, are similarly associated with NSSI in men.

Past research on NSSI and gender has focused on differences between women and men (Bresin & Schoenleber, 2015; Victor et al., 2018; Whitlock et al., 2011). This emphasis on gender differences is dominant across disciplines (Hyde, 2014), however, alternative frameworks exist. Our findings are consistent with Hyde’s (2005) *Gender Similarities Hypothesis* which asserts that women and men are similar across most psychological domains, including their experience of emotion. Our findings also parallel conclusions that “the similarities between men and women in emotion regulation are greater than the differences” (Nolen-Hoeksema, 2012, p. 176), and that men and women do not meaningfully differ on most NSSI characteristics (Victor et al., 2018).

### Age Effects

Our results are consistent with meta-analytic findings that age does not moderate the gender prevalence gap in early adulthood (Bresin & Schoenleber, 2015). However, all participants were aged at least 18, so these results may not generalize to adolescents. Contrary to expectation, past-year NSSI prevalence did not significantly decrease with age, and participants here disclosed much higher lifetime and past-year NSSI rates than those reported in past studies of young adults (Moran et al., 2012; Swannell, Martin, Page, Hasking, & St John, 2014; Taliaferro & Muehlenkamp, 2015). Endorsement of NSSI is thought to be highest in mid-adolescence before declining into young adulthood (Daukantaite et al., 2021; Plener et al., 2015) leading to assertions that “most self-harming behavior in adolescents resolves spontaneously” (Moran et al., 2012, p. 236). The true scope of NSSI among young adults and the reason for discrepancies remain unclear.

Some participants may have previously ceased NSSI but returned to this behavior to cope with worsening mental health during the COVID-19 pandemic. Both clinical and community samples show increasing psychiatric symptoms and psychological distress during the pandemic, more so for women than men (Vindegaard & Benros, 2020; Wang et al., 2020). Experts have expressed concern that the reduction of face-to-face mental health services and accumulation of risk factors will lead to a “steep increase in NSSI” (Plener, 2021). However, we found no evidence of such an increase in this cohort—compared to data collected during a separate NSPN follow-up in 2017–2018, past-year NSSI prevalence here was numerically (though not statistically significantly) lower. Longitudinal research examining the impact of the pandemic on NSSI is needed.

### Limitations

The data reported here are cross-sectional and so we cannot demonstrate true mediation, but rather statistical mediation. Longitudinal associations between NSSI and the variables considered here are likely complex and bi-directional, as shown in previous waves of NSPN data (Cassels, Neufeld, van Harmelen, Goodyer, & Wilkinson, 2020; Polek et al., 2020). The non-significant association between age and prevalence, despite rates appearing to decline (Figure 2), may mean that analyses were underpowered to detect a significant effect. Our measures did not delineate NSSI or distress before versus during the COVID-19 pandemic and are limited by the exclusive use of self-report questionnaires. This study had only a 10-item scale of psychological distress, with fewer items and psychological constructs than other similar studies; it therefore may be a less accurate measure of distress than other studies (e.g., Wilkinson et al., 2022). However, past research has demonstrated good validity and precision (at the clinical range) of the K10 (Kessler et al., 2002), suggesting it is an adequate measure.

Gendered assumptions about NSSI may result in underreporting of NSSI among men (Green & Jakupcak, 2016; Kimbrel, Calhoun, & Beckham, 2017). Greater endorsement of psychological distress could reflect women’s conformity to gender role expectations or a greater tendency to self-disclose mental-health symptoms (Nolen-Hoeksema, 2012). However, men and women respond similarly on the K10 (Baillie, 2005; Kessler et al., 2002), and gender differences in depression levels are unlikely to result from gender-biased responding (Hyde & Mezulis, 2020).

The present study is limited by our binary classification of gender. We do not know how many participants are transgender and analyses excluded seven participants who reported their gender identity as “other.” Future research should adopt more accurate measures of gender (Hyde, Bigler, Joel, Tate, & van Anders, 2019) and include gender non-conforming participants, who are at higher risk of NSSI (Liu et al., 2019).

### Future Directions

Greater gender parity in NSSI research is needed. Research on gender similarities and differences in risk factors, functions, and progression of NSSI are necessary for development of interventions which support NSSI recovery in people of all genders. Future research should consider variables which affect the relationships between risk factors and NSSI. The literature on mediators and moderators is limited, with many studies revealing conflicting results or needing replication (Valencia-Agudo et al., 2018). Research is especially needed in adult samples. Furthermore, beyond identifying correlates of NSSI, the literature should investigate how etiologic factors relate to the experience of NSSI. A recent study by Lockwood et al. (2020) is one such example, utilizing qualitative methods to explore the cognitive and emotional factors involved in the build-up to acts of self-harm. The researchers demonstrate that people who engage in self-harm can offer valuable insights into the processes underlying their behavior and underscore that there is wide variation in the experience of NSSI both within and between individuals.

Clinicians should be aware of how common NSSI is among young adults. Findings here reveal potential avenues for intervention, including developing strategies which reduce psychological distress or improve distress tolerance, strengthen emotion regulation skills and control impulsive reactions, and replace NSSI with other coping strategies. These are potential therapeutic targets for interventions such as Dialectical Behavioral Therapy (DBT), which emphasizes these skills and effectively reduces self-harm in adolescents and adults (Kothgassner, Robinson, Goreis, Ougrin, & Plener, 2020; Lynch, Trost, Salsman, & Linehan, 2007). However, the strength of the evidence for DBT is limited as randomized control trials have primarily recruited women (DeCou, Comtois, & Landes, 2019; Wilkinson, 2018). Given our results demonstrate that men and women do not differ on how similar levels of psychological distress, emotion dysregulation, or impulsivity relate to NSSI, it is possible that the same treatments work for men and women. However, this requires testing within treatment studies; importantly these studies need to include sufficient participant numbers across gender groups.

## Conclusion

Results demonstrate that women are more likely to report NSSI due, in part, to their significantly higher levels of psychological distress than men. We show that given similar levels of psychological distress, emotion dysregulation, and impulsivity, women and men are similarly likely to experience NSSI. Our findings extend literature on gender differences in depressive symptoms to explain the NSSI prevalence gap and support Hyde’s *Gender Similarities Hypothesis*. Future research should continue to investigate similarities and differences in NSSI across genders.

## Figures and Tables

**Table 1:** Univariate associations between gender/past-year NSSI and self-reported psychological distress, emotion dysregulation, and impulsivity.

	Gender				Past-year NSSI			
	Women *M(SD)*	Men *M(SD)*	Coef. [95% Cl]	*p*	Yes *M(SD)*	No *M(SD)*	Coef. [95% Cl]	*p*
Psychological distress^a^	22.54 (7.40)	20.44 (7.59)	2.10 [1.13, 3.07]	**<.001**	29.94 (7.64)	20.65 (6.80)	0.16 [0.13, 0.19]	**<.001**
Emotion dysregulation^b^	38.33 (11.67)	36.82 (11.82)	1.51 [−0.09, 3.11]	.065	49.59 (12.62)	36.02 (10.56)	0.09 [0.07, 0.11]	**<.001**
Negative urgency^c^	8.60 (2.68)	8.68 (2.56)	−0.08 [−0.43, 0.26]	.64	10.68 (2.54)	8.35 (2.52)	0.36 [0.27, 0.44]	**<.001**
Lack of perseverance^c^	8.38 (1.80)	8.25 (1.71)	0.13 [−0.10, 0.36]	.28	8.30 (2.00)	8.34 (1.74)	-0.01 [−0.12,0.10]	.81
Lack of premeditation^c^	7.89 (0.07)	7.85 (0.10)	0.04 [−0.20, 0.27]	.75	8.41 (1.83)	7.81 (1.77)	0.19 [0.08, 0.29]	**.001**
Sensation seeking^c^	8.91 (2.50)	10.89 (2.50)	−1.98 [−2.30, −1.65]	**<.001**	9.12 (2.17)	9.70 (2.66)	−0.08 [−0.16, −0.01]	**.029**
Positive urgency^c^	6.77 (2.17)	7.59 (2.41)	-0.83 [−1.12, −0.53]	**<.001**	8.22 (2.59)	6.91 (2.21)	0.23 [0.15, 0.31]	**<.001**

Bold indicates significance at *p* < .05. ^a^Kessler Psychological Distress Scale (K10). ^b^Difficulties in Emotion Regulation Scale (DERS) short form. ^c^UPPS-P impulsivity questionnaire short form.

**Table 2:** Age and gender effects on past-year NSSI prevalence.

	OR	95% CI	*p*
Gender	2.00	1.29, 3.13	**.002**
Age	0.96	0.90, 1.02	.20
Age^2^	1.00	0.98, 1.02	.91
Age × Gender	1.02	0.88, 1.18	.80
Age^2^ × Gender	1.00	0.96, 1.05	.98

Bold indicates significance at *p* < .05.

## Data Availability

Data are available upon reasonable request to the corresponding author.

## Highlights

- Women aged 18–33 were significantly more likely to report pastyear NSSI than men
- Women’s greater psychological distress contributed to their higher NSSI prevalence
- Variables investigated here were similarly associated with NSSI in men and women
